# Investigation of the Substrate Selection Mechanism of Poly (A) Polymerase Based on Molecular Dynamics Simulations and Markov State Model

**DOI:** 10.3390/ijms26199512

**Published:** 2025-09-29

**Authors:** Yongxin Jiang, Xueyan Duan, Jingxian Zheng, Fuyan Cao, Linlin Zeng, Weiwei Han

**Affiliations:** Key Laboratory for Molecular Enzymology and Engineering of Ministry of Education, Edmond Fischer Cell Signaling Laboratory, School of Life Sciences, Jilin University, Changchun 130012, China

**Keywords:** gaussian accelerated molecular dynamics simulation, markov state model, molecular docking, substrate selectivity, RNA polymerase

## Abstract

RNA polymerases are essential enzymes that catalyze DNA transcription into RNA, vital for protein synthesis, gene expression regulation, and cellular responses. Non-template-dependent RNA polymerases, which synthesize RNA without a template, are valuable in biological research due to their flexibility in producing RNA without predefined sequences. However, their substrate polymerization mechanisms are not well understood. This study examines Poly (A) polymerase (PAP), a nucleotide transferase superfamily member, to explore its substrate selectivity using computational methods. Previous research shows PAP’s polymerization efficiency for nucleoside triphosphates (NTPs) ranks ATP > GTP > CTP > UTP, though the reasons remain unclear. Using 500 ns Gaussian accelerated molecular dynamics simulations, stability analysis, secondary structure analysis, MM-PBSA calculations, and Markov state modeling, we investigate PAP’s differential polymerization efficiencies. Results show that ATP binding enhances PAP’s structural flexibility and increases solvent-accessible surface area, likely strengthening protein–substrate or protein–solvent interactions and affinity. In contrast, polymerization of other NTPs leads to a more open conformation of PAP’s two domains, facilitating substrate dissociation from the active site. Additionally, ATP binding induces a conformational shift in residues 225–230 of the active site from a loop to an α-helix, enhancing regional rigidity and protein stability. Both ATP and GTP form additional π–π stacking interactions with PAP, further stabilizing the protein structure. This theoretical study of PAP polymerase’s substrate selectivity mechanisms aims to clarify the molecular basis of substrate recognition and selectivity in its catalytic reactions. These findings offer valuable insights for the targeted engineering and optimization of polymerases and provide robust theoretical support for developing novel polymerases for applications in drug discovery and related fields.

## 1. Introduction

RNA polymerase (RNAP), also known as transcriptase, is a multi-subunit protein complex that utilizes four nucleoside triphosphates (NTPs: ATP, GTP, CTP, and UTP) as substrates and DNA as a template to synthesize RNA through phosphodiester bond formation, thereby converting genetic information encoded in DNA into RNA [1]. RNAP is evolutionarily conserved across archaea, bacteria, and eukaryotes, and is responsible for the transcription of various RNA types. Within biological systems, RNAP is indispensable for transcription and gene expression regulation, initially transcribing double-stranded DNA into single-stranded RNA, which subsequently serves as a template for translation to produce polypeptide chains [2]. These polypeptides undergo further post-translational modifications and folding to form functionally active proteins [3]. Consequently, RNAP is not only a critical enzyme in gene expression but also fundamental to basic cellular processes, playing an essential role in cellular function, development, and adaptation to environmental changes [4].

Non-template-dependent RNA polymerases are a class of enzymes capable of synthesizing or modifying RNA directly from NTPs (ATP, UTP, CTP, and GTP) without requiring a DNA template, exhibiting efficient and unique catalytic properties [5]. These enzymes are significant in processes such as gene expression regulation, RNA modification, and virology [6]. Therefore, investigating their substrate incorporation mechanisms is crucial for understanding substrate specificity and catalytic mechanisms.

Poly(A) polymerase (PAP), a representative member of the nucleotidyltransferase superfamily, is a non-template-dependent RNA polymerase [7]. Its crystal structure reveals three globular domains: the N-terminal catalytic domain (NTD), the middle domain, and the C-terminal domain (CTD) [8]. PAP plays a pivotal role in post-transcriptional modification processes, particularly in mRNA polyadenylation [9]. Studies by Balbo et al. have demonstrated that, in the presence of all four NTP substrates (ATP, UTP, CTP, and GTP), PAP exhibits a significantly higher preference for incorporating purines over pyrimidines. Further investigations revealed that PAP’s catalytic efficiency for GTP incorporation is approximately 800-fold lower than for ATP, with even lower efficiencies for CTP and UTP [10]. Thus, the substrate incorporation efficiency of PAP follows the order ATP > GTP > CTP > UTP. However, the molecular details of this incorporation kinetics, particularly the mechanisms underlying substrate selectivity, remain unclear and require further exploration.

Molecular dynamics (MD) simulations are computational techniques based on classical mechanics principles, designed to study the structure and interactions of molecular systems [11]. With advancements in molecular force fields and high-performance computing, MD simulations have become a powerful tool for investigating the dynamic properties, structural changes, thermodynamic characteristics, and molecular interactions of biomolecular systems [12]. MD simulations provide time–evolution trajectories of molecular systems under various conditions, offering insights into the relationship between molecular structure and function [13].

Gaussian Accelerated Molecular Dynamics (GaMD) is an advanced method to enhance the efficiency of traditional MD simulations [14]. As an effective enhanced sampling technique, GaMD introduces a smooth, random Gaussian-distributed bias potential to the system’s potential energy surface, reducing free energy barriers [15]. This facilitates more efficient exploration of a broader potential energy landscape and promotes rapid barrier crossing [16]. GaMD enables researchers to extract critical dynamic features, such as time constants and spectral line shapes, with higher precision from experimental data [17]. It also allows for the acquisition of richer dynamic information in shorter simulation times and provides accurate free energy distributions through reweighting [18].

This study employs GaMD simulations to systematically analyze the substrate selectivity of two representative non-template-dependent RNA polymerases, PAP and Cid1, when catalyzing different NTPs (ATP, UTP, CTP, and GTP). Through MD simulations, we investigate enzyme–substrate interactions and their impact on enzyme structure and function, elucidating the molecular mechanisms governing substrate incorporation. These findings provide a theoretical foundation for the design of RNA polymerases.

## 2. Results and Discussion

### 2.1. Binding of Four Ligand Molecules to PAP Polymerase

**Figure 1:**
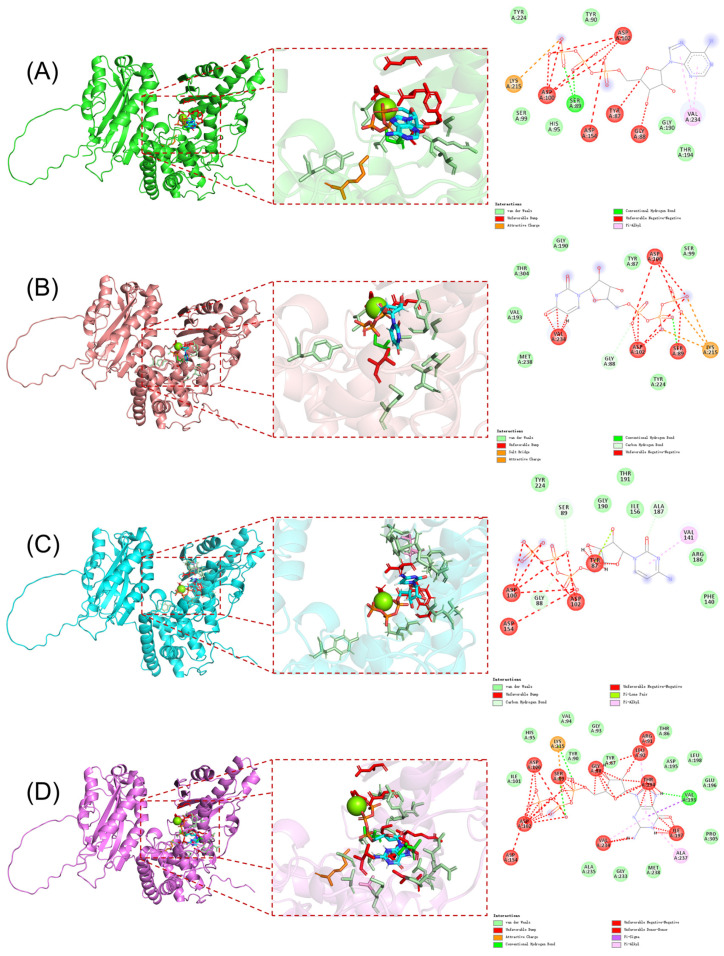
(**A**) Docking results of ATP with PAP protein; (**B**) Docking results of UTP with PAP protein; (**C**) Docking results of CTP with PAP protein; (**D**) Docking results of GTP with PAP protein.

After obtaining the crystal structure of PAP polymerase from the PDB database, molecular docking of the PAP protein with four NTP ligand molecules was performed using AutoDock Vina 1.1.2 software. The docking results of the PAP protein with the four ligand molecules are shown in Figure 1, which also displays the amino acid residues interacting with the protein. Comparative analysis reveals that when ATP and GTP are used as substrates, the number of interacting residues between the ligand molecules and the protein is significantly higher than when CTP and UTP are used as substrates. Further analysis indicates that Ser89 and Lys215 of the PAP protein form two hydrogen bonds with ATP, whereas these residues form only one hydrogen bond with CTP and GTP. This suggests that these two amino acid sites may play a critical role in the specific binding of ATP to the PAP protein by forming a stable hydrogen bond network, potentially explaining the differences in PAP’s polymerization efficiency with ATP, CTP, and GTP.

### 2.2. Protein Structure Stability Analysis

**Figure 2:**
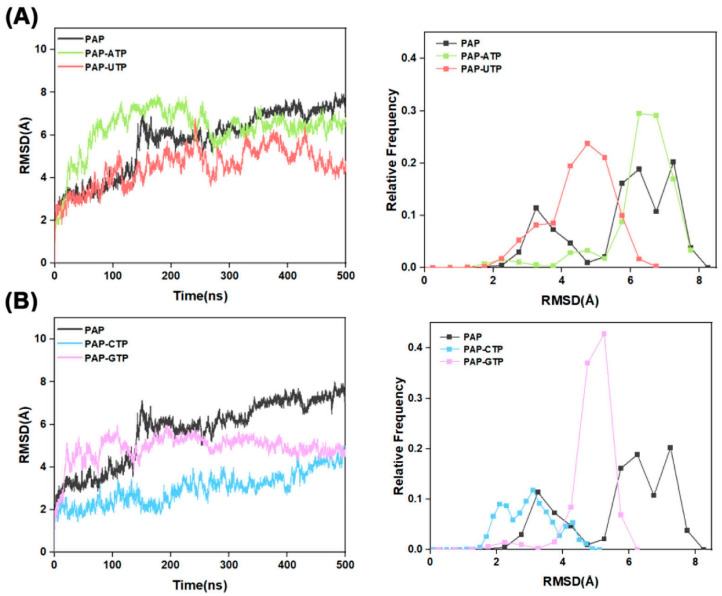
(**A**) RMSD plots for the systems Free-PAP, PAP-ATP, and PAP-UTP. The left panel depicts the variation of RMSD with simulation time, while the right panel illustrates the distribution of RMSD values; (**B**) RMSD plots for the systems Free-PAP, PAP-CTP, and PAP-GTP. The left panel depicts the variation of RMSD with simulation time, while the right panel illustrates the distribution of RMSD values.

To evaluate the structural stability of the Free-PAP, PAP-ATP, PAP-UTP, PAP-CTP, and PAP-GTP systems, this study conducted computational analyses of the root mean square deviation (RMSD), radius of gyration (R_g_), and solvent-accessible surface area (SASA) based on the Cα atom trajectory behavior during 500 ns GaMD simulations. The results are presented as follows:

RMSD serves as a pivotal metric in molecular dynamics simulations for evaluating the dynamic stability of protein–ligand complexes. As shown in Figure 2, a comparative evaluation indicates that the RMSD profiles of the ligand-bound systems are lower than those of the apo-protein, signifying enhanced rigidity in the surrounding structural motifs and, consequently, augmented overall protein stability. This observation further implies that the binding of the four NTPs bolsters enzymatic stability. Additionally, the rapid stabilization of the ATP trajectory, achieving equilibrium within approximately 100 ns, underscores its superior polymerization efficiency; the GTP curve exhibits moderate equilibration around 150 ns, with RMSD ~3 Å and a moderate distribution; whereas the prolonged equilibration times for UTP and CTP, approximately 200 ns, with RMSD values of 4–5 Å, broad distributions, and high fluctuations, align with their lower efficiencies and propensities for substrate dissociation, in strong concordance with the NTP selectivity ranking ATP > GTP > CTP > UTP.

### 2.3. Radius of Gyration Analysis

**Figure 3:**
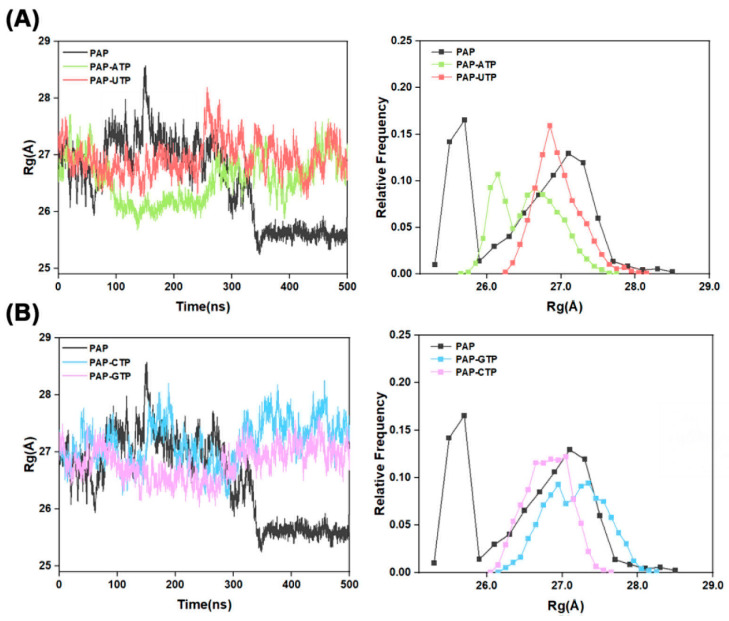
(**A**) R_g_ plots for the systems Free-PAP, PAP-ATP, and PAP-UTP; (**B**) R_g_ plots for the systems Free-PAP, PAP-CTP, and PAP-GTP. The left panel depicts the variation of R_g_ with simulation time, while the right panel illustrates the distribution of R_g_ values.

The radius of gyration (R_g_) is a key physical parameter used to measure the compactness of a biomacromolecule’s spatial structure, commonly applied to study molecular conformation and flexibility. As shown in Figure 3, when the four different NTPs are used as substrates, the R_g_ values for the PAP-ATP, PAP-UTP, PAP-CTP, and PAP-GTP systems fluctuate around 26.56 Å, 26.96 Å, 27.17 Å, and 26.81 Å, respectively. Comparative analysis indicates that the R_g_ distributions of the PAP-ATP and PAP-GTP systems are more concentrated than those of the other two systems, suggesting that these two systems occupy a smaller volume in space and exhibit a more compact structure. Furthermore, during the simulation process, the possible reason for the significant decrease in the radius of gyration (R_g_) of PAP—from 27 to 25.5—could be that the initial protein structure we selected was a conformation of the protein bound to the substrate; however, in the molecular dynamics simulation process, there was no substrate in the entire system. Upon losing the induction from the substrate, it will automatically transition to a natural compact conformation, which is why there was a significant drop in R_g_ at a certain moment.

### 2.4. Solvent-Accessible Surface Area Analysis

**Figure 4:**
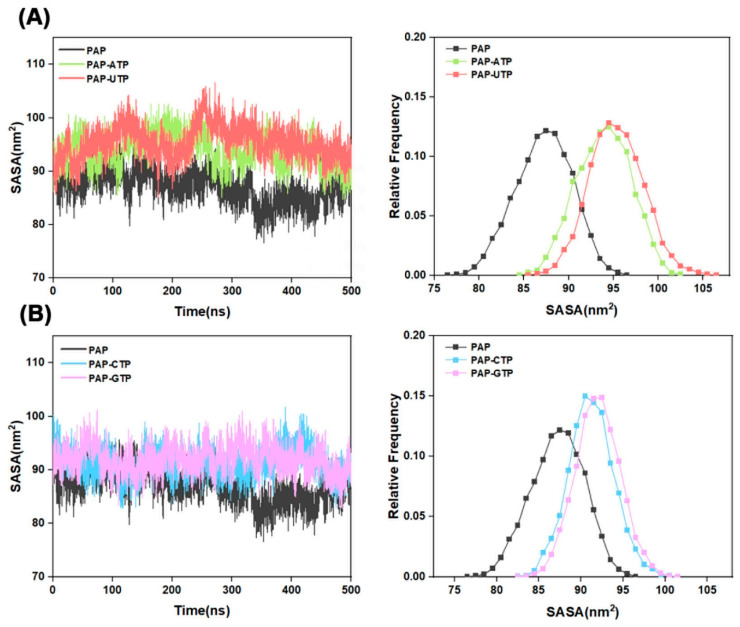
(**A**) SASA plots for the systems Free-PAP, PAP-ATP, and PAP-UTP; (**B**) SASA plots for the systems Free-PAP, PAP-CTP, and PAP-GTP. The left panel depicts the variation of SASA with simulation time, while the right panel illustrates the distribution of SASA values.

The solvent-accessible surface area (SASA) is a critical parameter for evaluating the interaction between a protein and solvent molecules, reflecting the contact area of the molecular structure with the solvent. As shown in Figure 4, compared to the apo-protein group, the SASA values of the four ligand-bound systems exhibit significant differences, indicating that ligand binding induces conformational changes in the protein, exposing a larger surface area and increasing the hydrophilic contact area with the solvent. Notably, the SASA fluctuations in the PAP-ATP system are greater than those in the other three systems, suggesting that the binding of ATP to the protein significantly increases the contact area between the protein and the solvent, while also markedly affecting its hydrophilicity.

### 2.5. Temperature Factor Analysis

**Figure 5:**
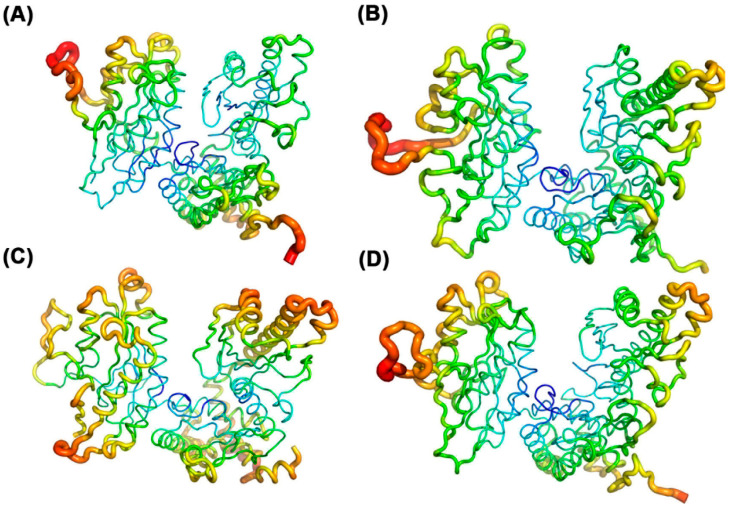
Temperature factor(b-factor) analysis of four systems: (**A**) PAP-ATP; (**B**) PAP-UTP; (**C**) PAP-CTP; (**D**) PAP-GTP. Color red indicates higher B-factor values, green indicates intermediate B-factor values, and blue indicates lower B-factor values, representing flexibility and stability respectively.

The B-factor (also known as the Debye–Waller factor or temperature factor) characterizes the scattering effect caused by atomic thermal motion in X-ray diffraction experiments, reflecting the thermal stability of atoms within a protein structure. In molecular dynamics (MD) simulations, the B-factor value (B-value) is used to assess the flexibility and mobility of atoms, amino acid side chains, and loop regions in a protein. A higher B-value indicates greater mobility and flexibility in a given region, while a lower B-value suggests higher rigidity and stability [19]. As shown in Figure 5, blue regions represent lower B-values, indicating higher stability, whereas red regions indicate higher B-values, reflecting greater fluctuations. The catalytically active sites of the four protein systems bound to ATP, UTP, CTP, and GTP all exhibit low B-value regions, suggesting that these areas are relatively rigid and that substrate binding stabilizes the protein’s active site, consistent with previous analyses. Further analysis reveals that when ATP is the substrate, the B-factor plot shows a higher proportion of blue regions in the active site, indicating that ATP stabilizes the protein structure and reduces the flexibility of the active site through its interactions with the protein. This stability likely stems from the strong affinity of ATP for the protein, forming a stable binding site that reduces the mobility and vibrational amplitude of the active site. This further demonstrates that ATP binding enhances the overall structural stability of the protein.

### 2.6. Binding Free Energy Calculation

Binding free energy is commonly used to describe the free energy change associated with the protein–ligand binding process, reflecting the strength of their interaction, which is typically directly correlated with ligand potency. Table 1 lists the binding free energies between PAP and the ligands in the four systems, where ∆E_vdW_ represents the van der Waals energy term, ∆G_solv_ denotes the solvation energy, and ∆G_gas_ corresponds to the molecular mechanics term (energy in the gas phase). Specifically, the total binding free energy (∆G_total_) for the PAP-ATP system is −753.76 kcal/mol, significantly lower than that of the other three systems, indicating a much higher affinity of ATP for PAP and its critical role in stabilizing the protein’s conformation. The binding free energy (∆G_total_) for the PAP-GTP system is −410.94 kcal/mol, slightly lower than that of the other two systems, suggesting a relatively strong affinity between the protein and the ligand.

Additionally, the van der Waals interaction energies (∆E_vdW_) for the PAP-ATP, PAP-UTP, and PAP-CTP systems are lower than their electrostatic interaction energies (∆E_ele_), indicating that van der Waals forces dominate the binding free energy in these three systems. In contrast, in the PAP-GTP system, the electrostatic interaction energy plays the primary role.

**Figure 6:**
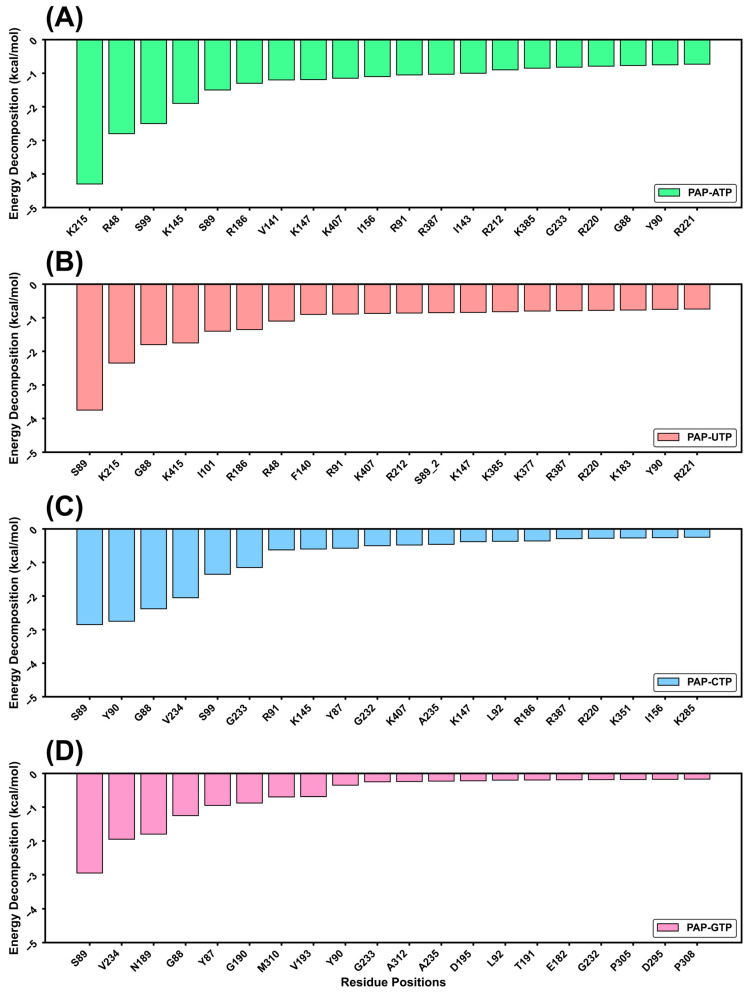
The 20 residues in the four system complexes that contribute most to the free energy: (**A**) PAP-ATP; (**B**) PAP-UTP; (**C**) PAP-CTP; (**D**) PAP-GTP.

Next, the binding free energy was decomposed into contributions from individual residues, as shown in Figure 6. The results reveal that the binding energy magnitudes follow the order: PAP-ATP > PAP-GTP > PAP-CTP > PAP-UTP. This difference is hypothesized to be closely related to the role of the Lys215 residue in the active site. These findings collectively indicate that, in the presence of the four substrates, the selectivity of PAP for the substrates follows the order: PAP-ATP > PAP-GTP > PAP-CTP > PAP-UTP.

### 2.7. Salt Bridge Analysis

Following the preliminary investigation of the mechanisms underlying the PAP protein’s differential substrate selectivity through analyses of stability, secondary structure changes, and cross-correlation, we further conducted a salt bridge analysis to gain deeper insights into the structural changes induced by the binding of different ligands. As shown in Table 2, comparative analysis reveals that the salt bridge formation rate in the PAP-ATP complex is 0.028, slightly higher than that in the PAP-GTP complex at 0.024. This result corroborates the trend of PAP-ATP > PAP-GTP > PAP-CTP > PAP-UTP. We hypothesize that these salt bridges play a critical role in the functional adjustments of the enzyme, ensuring its adaptability upon substrate binding.

### 2.8. π–π. Stacking Analysis

π–π stacking interactions play a significant role in enhancing binding affinity and specificity. Given that all four nucleotide ligands possess aromatic ring structures, this study investigated the π–π stacking interactions between the protein and ligands. As shown in Table 3, the π–π stacking occupancy rates for the four systems follow the order: PAP-ATP > PAP-GTP > PAP-UTP > PAP-CTP. This trend further indicates that the binding stability between the protein and ligands is directly correlated with π–π stacking interactions.

### 2.9. Dynamic Cross-Correlation Matrix (DCCM) Analysis

Using the Bio3d package in R, we calculated the DCCM for protein atoms based on molecular dynamics simulation trajectories. In the generated matrix, the color intensity represents the degree of correlation: deep blue indicates positive correlation, deep pink indicates negative correlation, and white indicates no correlation (i.e., independent motion of the two atoms).

**Figure 7:**
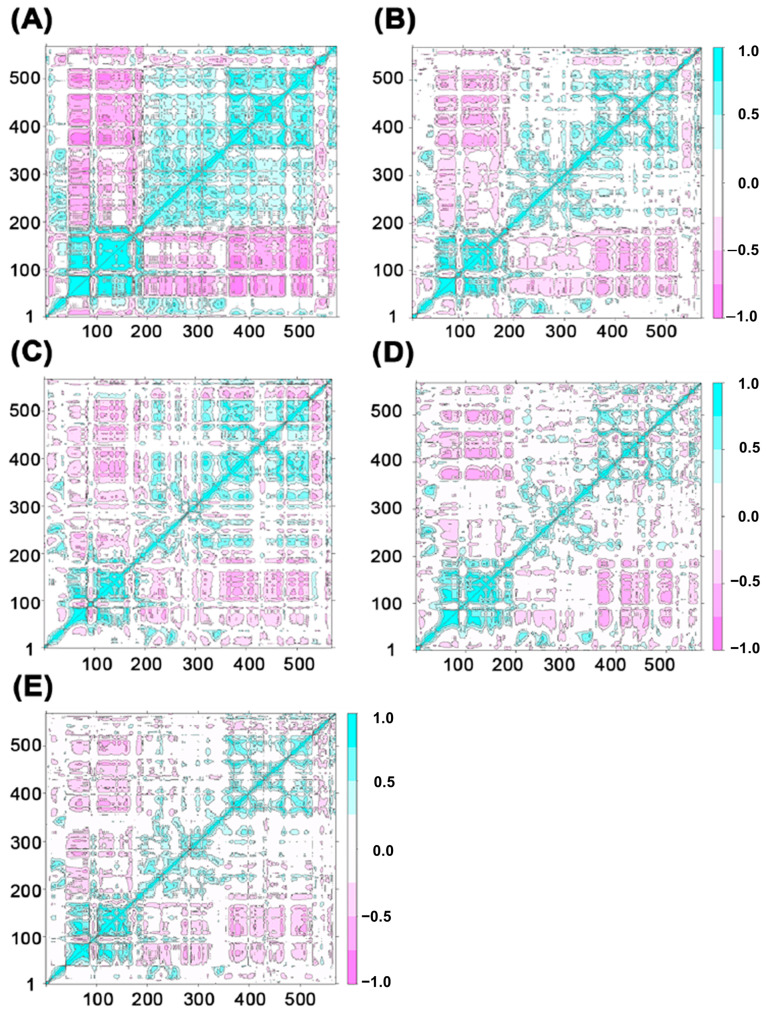
Cross-Correlation Analysis (CCA) of five systems: (**A**) PAP; (**B**) PAP-ATP; (**C**) PAP-UTP; (**D**) PAP-CTP; (**E**) PAP-GTP.

Figure 7 displays the DCCM matrices for all Cα atoms in the five systems. The results show that, compared to the ligand-free PAP, the flexibility of the system decreases upon ligand binding. Notably, the DCCM plot for the PAP-ATP system exhibits the most prominent blue regions, indicating a strong positive correlation in the motion of atoms within the complex. This suggests that these regions maintain consistent conformations during the simulation, resulting in overall greater stability. These findings confirm that ATP is the optimal substrate for the PAP protein, consistent with experimental results.

### 2.10. Protein Residue Flexibility Analysis

**Figure 8:**
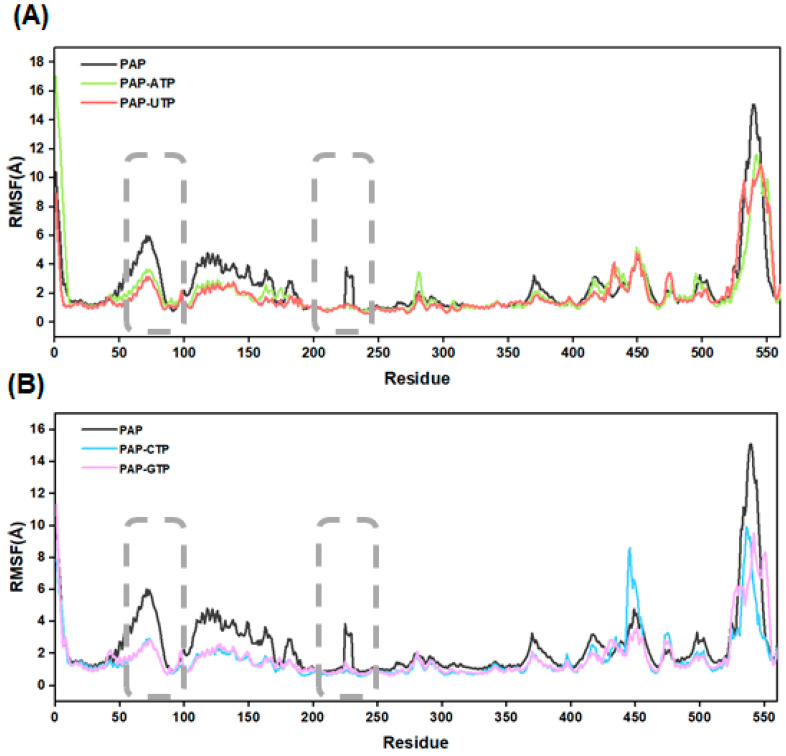
(**A**)RMSF plots for the systems Free-PAP, PAP-ATP, and PAP-UTP; (**B**) RMSF plots for the systems Free-PAP, PAP-CTP, and PAP-GTP.

To investigate the fluctuation of residues in the PAP polymerase, this study calculated the root mean square fluctuation (RMSF) values for the five systems (see Figure 8). The results indicate that during the 500 ns GaMD simulations, the apo-PAP protein exhibits slightly higher RMSF values overall compared to the other four systems, suggesting that ligand binding enhances the overall stability of the protein structure. Further analysis reveals that in all five systems, significant atomic fluctuations are observed in residues 81–87 and 225–230. Both segments are located near the protein’s active site, indicating that the secondary structure surrounding the active site displays high adaptability during substrate polymerization. For instance, in the PAP-ATP system, residues 225–230 transition from a loop structure to an α-helix, and this structural rearrangement facilitates more effective substrate recognition and binding by the enzyme.

### 2.11. Secondary Structure Changes of the Protein During Simulation

**Figure 9:**
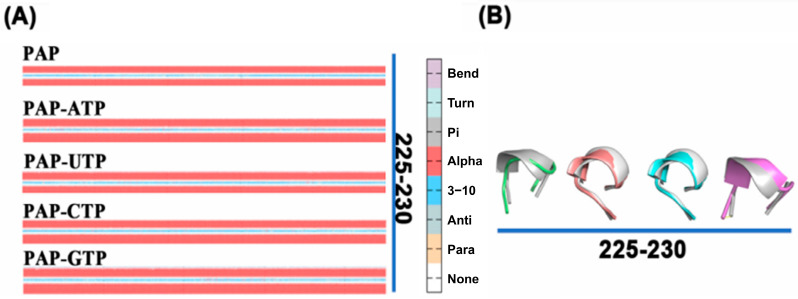
(**A**) DSSP changes in the five systems; (**B**) Secondary structure of the G81-Y87 region, Free-PAP (silver grey), PAP-ATP (green), PAP-UTP (pink), PAP-CTP (blue), PAP-GTP (purple).

The conformation of a protein undergoes changes over time during molecular dynamics simulations. Building on the RMSF analysis, we further examined the secondary structure changes of residues 81–87 and 225–230 using the DSSP algorithm. As shown in Figure 9, compared to the apo-PAP protein, the β-sheet structure in residues 81–87 is significantly shortened when ATP is bound, indicating that ATP binding induces local structural rearrangement in the protein, making the active site more suitable for substrate binding or catalytic reactions. The shortening of the β-sheet may be an adaptation to the spatial requirements of the substrate, potentially stabilizing the active site by reducing the β-sheet region, thereby maintaining a more ordered conformation and providing a better binding site. For residues 225–230, the secondary structure transitions from an α-helix in the apo-PAP protein to a loop structure, suggesting that ATP binding increases the overall flexibility of the protein, facilitating ligand binding.

The analysis of secondary structure not only reveals local spatial conformations but also provides insights into active site information related to biological function. Based on the secondary structure analysis, it is hypothesized that the enhanced activity of the protein when ATP is the substrate may be attributed to changes in its local secondary structure, which differ from the conformational changes observed with other substrates.

### 2.12. Markov State Model (MSM) Analysis

**Figure 10:**
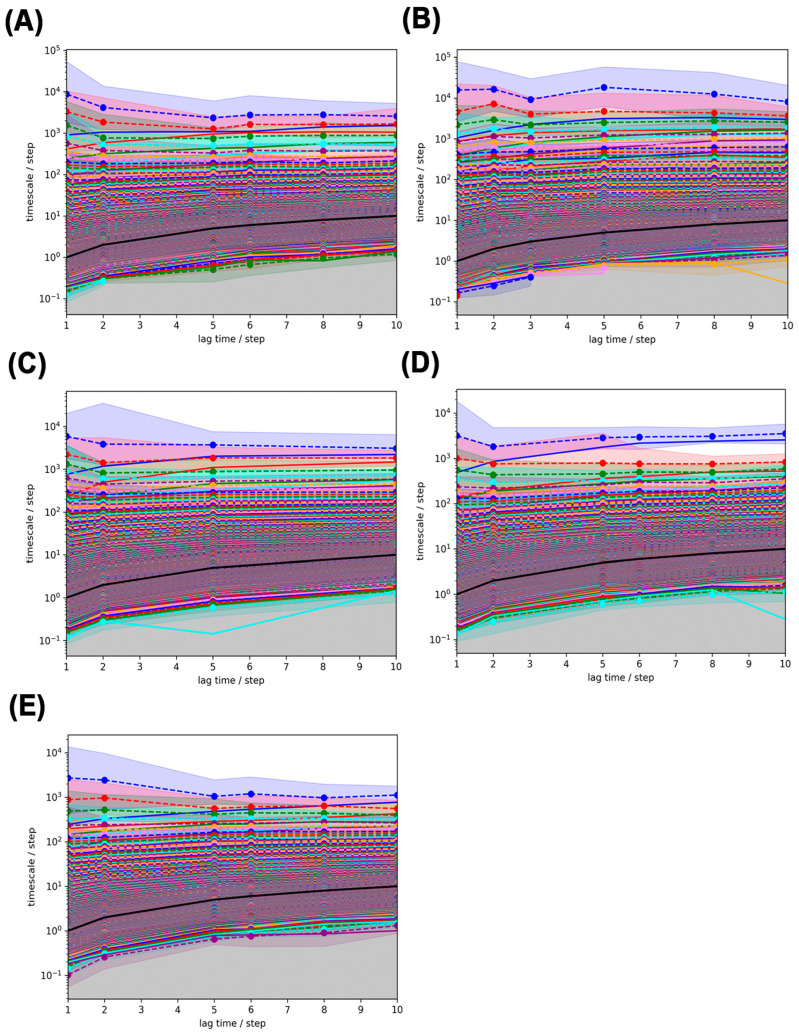
ITS Implied Time Scales plots for different simulated system MSMs (Implied Time Scales plots): (**A**) Free-PAP; (**B**) PAP-ATP; (**C**) PAP-UTP; (**D**) PAP-CTP; (**E**) PAP-GTP.

In this study, we refined the Markov State Model (MSM) by selecting an optimal lag time (τ), which is the time interval at which the system’s dynamics become independent of its past states (a key Markovian property). Figure 10 illustrates this process by plotting the implied timescales (ITS), which represent how long it takes for the system’s dynamics to stabilize, against different lag times. The black line in Figure 10 marks the threshold lag time (τ); ITS values above this line indicate independent and reliable dynamics. Our analysis showed that many ITS were longer than the chosen lag time, stabilizing after two steps, prompting us to adopt a lag time of 2 for constructing the MSM.

**Figure 11:**
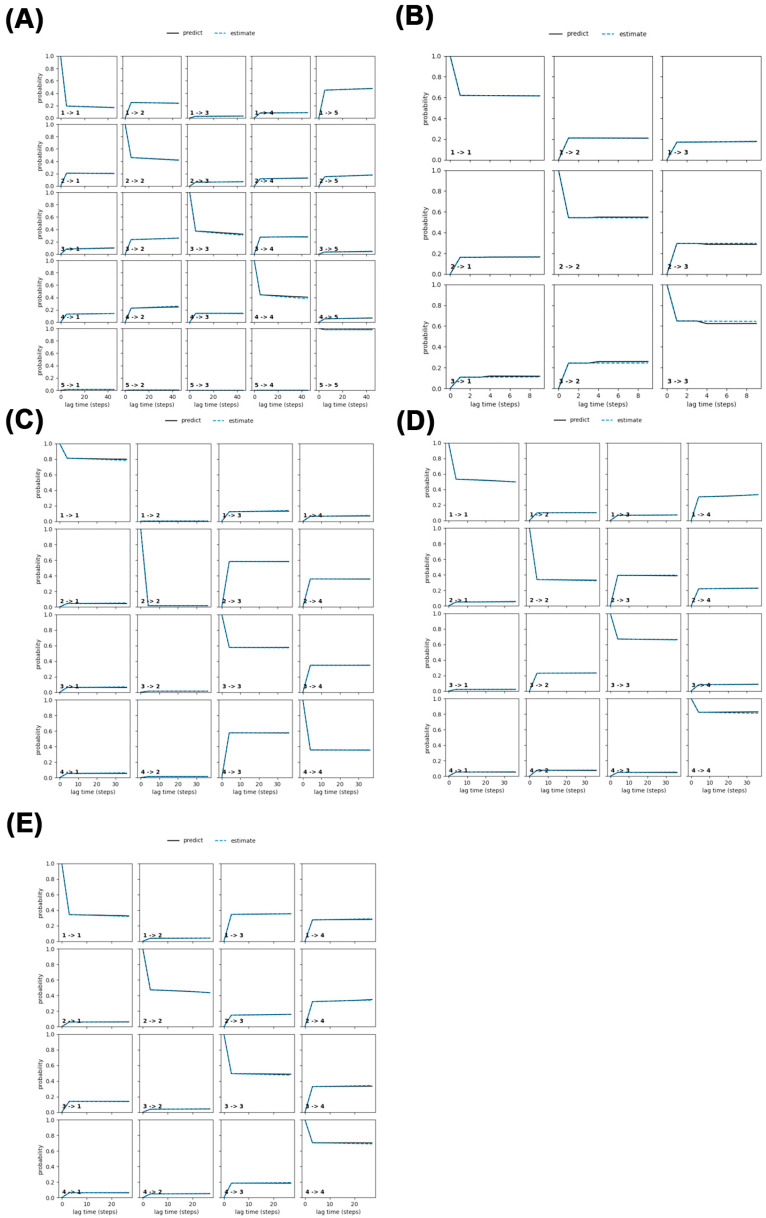
CK test for MSM of different simulation systems: (**A**) Free-PAP; (**B**) PAP-ATP; (**C**) PAP-UTP; (**D**) PAP-CTP; (**E**) PAP-GTP.

The system was divided into four distinct states to capture its dynamic behavior. To validate the MSM’s accuracy, we conducted the Chapman–Kolmogorov (CK) test, visualized in Figure 11. This test compares the MSM’s predictions (based on short lag times, shown as the black line) with observed probabilities at longer lag times (shown as the blue line). The close alignment between these lines across all systems confirms that the model accurately captures the system’s Markovian dynamics. Together, the results from Figure 10 and Figure 11 demonstrate the robustness and reliability of the MSM.

**Figure 12:**
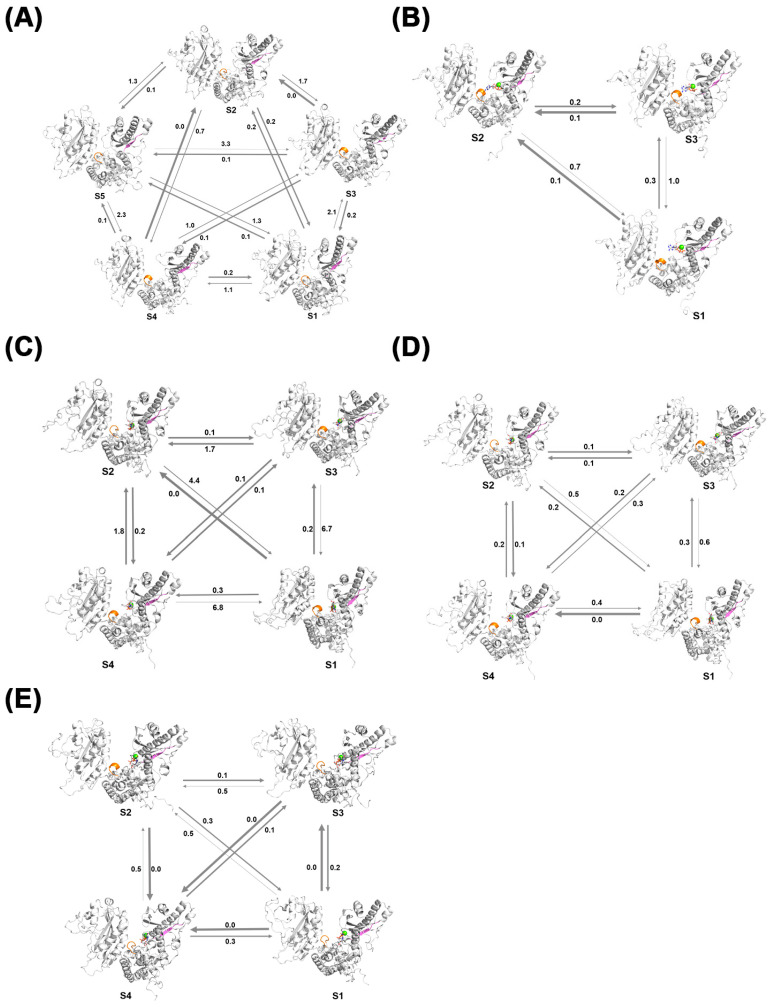
MSM of the five simulated systems: (**A**) Free-PAP; (**B**) PAP-ATP; (**C**) PAP-UTP; (**D**) PAP-CTP; (**E**) PAP-GTP. The parts displayed in special colors in the figure indicate regions where structural changes have occurred.

The Perron Cluster Cluster Analysis (PCCA) algorithm is a mathematical technique used to analyze clustering features in Markov chains. In this study, the PCCA method was employed to construct MSM models for the Free-PAP, PAP-ATP, PAP-UTP, PAP-CTP, and PAP-GTP systems. Figure 12A illustrates the MSM model for the apo-PAP system, where state S5 is a stable state, and the other four states are more likely to transition to S5. In Figure 12B, the MSM model for the PAP-ATP system shows state S2 as a stable state, with the other two states more likely to transition to S2, indicating that intermediate conformations readily transition to the dominant conformation, achieving a stable state more easily. Additionally, in terms of secondary structure, the 225–230 residues in the intermediate transition states are predominantly in an α-helix conformation, suggesting that ATP binding renders the active region of the PAP protein more rigid, thereby increasing the overall stability of the protein, consistent with previous findings. Figure 12C presents the MSM model for the PAP-UTP system, with state S3 as a stable state and the other three states more likely to transition to S3, with secondary structure changes aligning with prior analyses. Figure 12D shows the MSM model for the PAP-CTP system, where state S4 is a stable state, with the other three states more likely to transition to S4, and its secondary structure changes are consistent with previous results. Figure 12E displays the MSM model for the PAP-GTP system, with state S4 as a stable state and the other three states more likely to transition to S4, with secondary structure changes also consistent with prior analyses.

Comparative analysis of the systems reveals that the apo-PAP system exhibits more state transitions and is less likely to reach a stable state compared to the ligand-bound systems. This further confirms that ligand binding facilitates a more stable protein structure. Among the ligand-bound systems, the PAP-ATP system demonstrates faster state transitions and a greater propensity to reach a stable state, consistent with previous conclusions.

## 3. Materials and Methods

### 3.1. System Preparation

The crystal structure of yeast poly(A) polymerase (PAP) was retrieved from the RCSB Protein Data Bank (PDB ID: 2HHP) (https://www.rcsb.org/, accessed on 6 November 2024.) (Research Collaboratory for Structural Bioinformatics, Piscataway, NJ, USA) [10,20]. The structures of the four nucleoside triphosphates (NTPs: ATP, UTP, CTP, and GTP) were extracted from the NTP-bound complex (PDB ID: 3SV1). The protein was preprocessed using Discovery Studio 2021 (Dassault Systèmes BIOVIA, Vélizy-Villacoublay, France) to remove water molecules and ligands. Missing residues were modeled using MODELLER 10.2 (Sali Lab, University of California, San Francisco, San Francisco, CA, USA) [21]. Molecular docking of the four ligands (ATP, UTP, CTP, and GTP) to the receptor protein’s binding site was performed using AutoDock Vina 1.1.2 (Computational Chemical Sciences and Biology, The Scripps Research Institute, La Jolla, CA, USA), resulting in four systems: PAP-ATP, PAP-UTP, PAP-CTP, and PAP-GTP [22,23]. The MCPB.py (Merz Research Group, Michigan State University, East Lansing, MI, USA) tool was employed to handle metal ions and construct the metal ion force field [24]. Using Amber22, the amino acid residues of PAP were renumbered from 1 to 902 to finalize the setup of the simulation systems [25]. A total of five systems were constructed: System 1: ligand-free PAP; System 2: PAP bound to ATP; System 3: PAP bound to UTP; System 4: PAP bound to CTP; and System 5: PAP bound to GTP. In the latter four systems, Mg^2+^ ions were coordinated with oxygen atoms from water molecules.

### 3.2. Equilibrium Simulations

Five simulation systems were established for PAP: Free-PAP, PAP-ATP, PAP-UTP, PAP-CTP, and PAP-GTP. Conventional molecular dynamics (cMD) simulations were performed using the pmemd.cuda module of Amber22 (Amber Molecular Dynamics Developer Community, University of California, San Francisco, San Francisco, CA, USA). The protein force field parameters were generated using the Leap module of Amber, employing the ff19SB force field [26]. The four nucleotide ligands were parameterized using the GAFF2 force field. Each system was solvated in an octahedral box using the TIP3P water model, and counterions were added to ensure charge neutrality [27]. Non-bonded electrostatic interactions were calculated using the Particle Mesh Ewald (PME) algorithm with a cutoff radius of 8 Å [28]. Sodium ions were randomly added to neutralize the system’s charge. To minimize atomic clashes in the initial structures, energy minimization was performed for each system using 5000 steps of the steepest descent method followed by the conjugate gradient method. The systems were gradually heated to 300 K in the NVT ensemble and subsequently equilibrated for 50 ns in the NPT ensemble with a time step of 2 fs [29]. All files related to the molecular dynamics simulations are provided in the Supplementary Materials.

### 3.3. Gaussian Accelerated Molecular Dynamics (GaMD) Simulations

Following the 50 ns cMD simulations, 500 ns GaMD simulations were conducted for the Free-PAP, PAP-ATP, PAP-UTP, PAP-CTP, and PAP-GTP systems [30]. The ff19SB and GAFF2 force fields were used for the protein and ligand molecules, respectively. A water box with an 8 Å radius was created using the TIP3P water model to solvate the systems. Periodic boundary conditions were applied to minimize edge effects, and ions were added to neutralize the charge. A harmonic boost potential was applied to adjust the energy distribution, effectively lowering energy barriers to enhance sampling efficiency and accelerate transitions between system states. Based on parameters obtained from the prior 50 ns cMD simulations, 500 ns GaMD simulations were performed for each system in the NVT ensemble. Trajectories were analyzed to calculate root-mean-square deviation (RMSD), radius of gyration (R_g_), and solvent-accessible surface area (SASA) to assess system stability. Additionally, the Dictionary of Secondary Structure of Proteins (DSSP) was used to analyze secondary structure changes at key protein sites [31]. MM-PBSA residue binding energy analysis was performed to evaluate stability and conformational changes upon substrate binding [32,33]. Interactions such as salt bridges and π–π stacking between the protein and different ligands were compared to elucidate the molecular basis for ATP’s preferential binding.

### 3.4. Dynamic Cross-Correlation Analysis of Molecular Trajectories

The Dynamic Cross-Correlation Matrix (DCCM) analysis, by evaluating molecular dynamics simulation trajectories, calculates the cross-correlation functions between amino acid residues or atoms, effectively revealing the temporal dynamic characteristics of the system, such as vibrations, torsions, and deformations. This method provides a critical perspective for understanding the structural changes and stability of proteins during simulations.

### 3.5. Markov State Model (MSM) Construction

The Python 3.9.7 (Python Software Foundation, Wilmington, DE, USA) library PyEMMA 2.5.11 (Freie Universität Berlin, Berlin, Germany) was used to analyze MD simulation data and construct Markov State Models (MSMs) [34]. Simulation data capturing the system’s dynamic behavior were collected to serve as the basis for subsequent analysis. Trajectory data were processed using PyEMMA to extract key features for model construction. Clustering algorithms, such as K-means, were applied to group the data. Transition probabilities between states were calculated to build a transition matrix. The accuracy of the MSM was validated using the Chapman–Kolmogorov (CK) test. Further analysis, including the examination of lag times and other critical dynamic features, was performed using PyEMMA to gain deeper insights into the system’s dynamics.

## 4. Conclusions

Through comprehensive analysis, we identified that the differential polymerization capabilities of PAP across four substrates are primarily driven by several key factors. Firstly, compared to other groups, ATP binding enhances the structural flexibility of the PAP protein and increases the solvent-accessible surface area. Analysis of B-factors, cross-correlation matrices, and MM-PBSA calculations revealed that the binding affinity between ATP and PAP is stronger. In contrast, when bound to NTPs other than ATP, the two domains of PAP are more likely to open, facilitating substrate dissociation from the active site pocket. Additionally, Markov State Model (MSM) results indicate that the PAP-ATP system achieves a faster stabilization process compared to systems with other ligands, suggesting that ATP binding enables PAP to reach a stable state more rapidly. These findings underscore the critical role of ATP in PAP protein stability and functional execution. Secondly, in terms of secondary structure changes, the PAP-ATP system exhibits a transition in the active site region (residues 225–230) from a loop to an α-helix, which enhances the rigidity of this region and thereby increases overall structural stability. Lastly, the energetic advantage of ATP binding confers higher affinity and stability, further supporting the affinity hierarchy of PAP-ATP > PAP-GTP > PAP-UTP > PAP-CTP.

To deepen this analysis, we can reference the computational work of Schlick and Kirmizialtin on polymerase selectivity and fidelity, which highlight the roles of conformational selection and ground-state nucleotide binding, showing significant similarities with our findings. Schlick’s team conducted molecular dynamics simulations and transition path sampling analyses on DNA polymerase β (pol β), revealing that correct nucleotides induce rapid transitions from open to closed conformations, enhancing stability through “gating” residues and optimized active-site geometry (e.g., Mg^2+^ coordination), while incorrect nucleotides lead to conformational distortions and elevated energy barriers, promoting dissociation. This aligns with our observation of rapid stabilization and domain closure in the PAP-ATP system, suggesting that ATP, as the preferred substrate, may amplify affinity differences through a similar induced-fit mechanism. Similarly, Kirmizialtin’s work on HIV reverse transcriptase (HIV RT) used targeted milestone methods to simulate millisecond-scale conformational dynamics, finding that correct nucleotides promote thermodynamically favorable closed pathways, while incorrect nucleotides, despite rapid transitions, are prone to release due to long-range electrostatic instability. This is consistent with our MSM results, highlighting the role of conformational selection in PAP’s preference for ATP. However, Schlick and Kirmizialtin’s studies delve deeper into energy landscapes and transition state details, such as free energy barriers and hydrogen bond networks, which are not yet fully covered in our analysis of PAP. By integrating their insights, we can infer that PAP’s domain-opening mechanism may involve similar “gating” residues and electrostatic drivers, further explaining the molecular basis of the NTP affinity hierarchy. This not only strengthens our conclusions but also provides directions for future work, such as exploring the specific energy contributions of PAP’s active site and the dynamic pathways of secondary structure transitions through advanced MD simulations to more comprehensively elucidate the mechanisms of polymerase substrate specificity.

In summary, this study provides a detailed understanding of the substrate specificity and polymerization mechanisms of Poly(A) Polymerase (PAP) with respect to ATP, UTP, CTP, and GTP. Through systematic molecular dynamics simulations, we uncovered that ATP is the preferred substrate due to its higher binding affinity and superior catalytic efficiency, while UTP, CTP, and GTP exhibit distinct reaction rates and catalytic profiles. These simulations elucidated the dynamic conformational changes and molecular interactions governing PAP’s catalytic cycle, offering mechanistic insights into its substrate selectivity and polymerization efficiency. These findings not only deepen our understanding of PAP’s functional mechanisms but also have significant implications for polymerase engineering and drug discovery. By revealing the molecular basis of substrate specificity, our results can guide the rational design of engineered PAP variants with tailored substrate preferences, enabling enhanced control over RNA synthesis for biotechnological applications. Furthermore, the detailed characterization of PAP’s catalytic pocket and substrate interactions provides a foundation for designing small-molecule inhibitors or modulators targeting RNA polymerases. Collectively, these insights establish a robust theoretical framework for advancing RNA polymerase engineering and developing precision therapeutics, paving the way for innovative applications in RNA-based technologies and drug development.

## Figures and Tables

**Table 1:** Binding free energy MM-PBSA results (kcal/mol).

Systems	PAP-ATP	PAP-UTP	PAP-CTP	PAP-GTP
∆E_vdW_	−62.97 ± 1.16	−109.78 ± 1.85	−62.10 ± 0.74	−48.41 ± 1.93
∆G_solv_	569.83 ± 2.40	572.96 ± 3.94	251.40 ± 0.74	518.75 ± 6.74
∆G_gas_	−1323.59 ± 2.78	−963.72 ± 4.70	−648.80 ± 1.23	−929.69 ± 7.28
∆G_total_	−753.76 ± 1.50	−390.77 ± 2.11	−397.40 ± 0.89	−410.94 ± 2.58

**Table 2:** Salt bridge results for the four systems.

Systems	Salt Bridge
PAP-ATP	2.8
PAP-UTP	0.016
PAP-CTP	0.018
PAP-GTP	0.024

**Table 3:** π–π stacking results for the four systems.

Systems	Pi Stacking
PAP-ATP	0.84
PAP-UTP	0.04
PAP-CTP	0.00
PAP-GTP	0.37

## Data Availability

Data will be made available on request.

## Supplementary Materials

The following supporting information can be downloaded at: https://www.mdpi.com/article/10.3390/ijms26199512/s1.

## Abbreviations

The following abbreviations are used in this manuscript:

PAP	Poly (A) polymerase
NTPs	nucleoside triphosphates
RNAP	RNA polymerase
NTD	N-terminal catalytic domain
CTD	C-terminal domain
MD	Molecular dynamics
GaMD	Gaussian Accelerated Molecular Dynamics
PDB	Protein Data Bank
cMD	Conventional molecular dynamics
PME	Particle Mesh Ewald
RMSD	root-mean-square deviation
R_g_	radius of gyration
SASA	solvent-accessible surface area
DSSP	Dictionary of Secondary Structure of Proteins
DCCM	Dynamic Cross-Correlation Matrix
MSM	Markov State Model
CK	Chapman–Kolmogorov
B-value	B-factor value
∆E_vdW_	van der Waals energy term
∆G_solv_	solvation energy
∆G_gas_	molecular mechanics term (energy in the gas phase)
∆G_total_	total binding free energy
CCA	Cross-Correlation Analysis
RMSF	root mean square fluctuation
PCCA	Perron Cluster Cluster Analysis
